# Investigating the value of arterial spin labeling and intravoxel incoherent motion imaging on diagnosing nasopharyngeal carcinoma in T1 stage

**DOI:** 10.1186/s40644-020-00339-6

**Published:** 2020-08-28

**Authors:** Yujie Li, Xiaolu Li, Xiaoduo Yu, Meng Lin, Han Ouyang, Lizhi Xie, Yuqing Shang

**Affiliations:** 1 Department of Diagnostic Radiology, National Cancer Center/National Clinical Research Center for Cancer/Cancer Hospital, Chinese Academy of Medical Sciences and Peking Union Medical College, No17, Panjiayuannanli, Chaoyang District, Beijing, P.R. China 100021; 2 MR Research China, GE Healthcare, Beijing Beijing, P.R. China 100176; 3 Department of Chronic Disease Epidemiology, Yale School of Public Health, Yale University, New Haven, CT CT06510 USA

**Keywords:** Nasopharyngeal neoplasms, Magnetic resonance imaging, Perfusion imaging, Diffusion magnetic resonance imaging

## Abstract

**Background:**

To investigate the diagnostic value of arterial spin labeling (ASL) and intravoxel incoherent motion (IVIM) imaging in distinguishing nasopharyngeal carcinoma (NPC) in T1 stage from healthy controls (HC).

**Methods:**

Forty-five newly diagnosed NPC patients in the T1 stage and thirty-one healthy volunteers who underwent MR examinations for both 3D pseudo-continuous ASL (pCASL) and IVIM were enrolled in this study. The Mann-Whitney test was used to compare the mean values of blood flow (BF) derived from pCASL and IVIM derived parameters, including apparent diffusion coefficient (ADC), pure molecular diffusion (D), pseudo-diffusion coefficient (D*) and perfusion fraction (f) between NPC tumor and benign nasopharyngeal mucosa of HC. Receiver Operating Characteristic (ROC) was performed to determine diagnostic cutoff and efficiency. The correlation coefficients among parameters were investigated using Spearman’s test.

**Results:**

The NPC in the T1 stage showed higher mean BF, lower ADC, D, and f compared to benign nasopharyngeal mucosa (P < 0.001) with the area under curve of ROC of 0.742–0.996 (highest by BF). BF cutoff was set at > 36 mL/100 g/min; the corresponding sensitivity, specificity, and accuracy in differentiating NPC stage T1 from benign nasopharyngeal mucosa were 95.56% (43/45), 100% (31/31) and 97.37% (74/76), respectively. BF demonstrated moderate negative correlation with D* on HC (ρ [Spearman correlation coefficients] = − 0.426, *P* = 0.017).

**Conclusions:**

ASL and IVIM could reflect the difference in perfusion and diffusion between tumor and benign nasopharyngeal mucosa, indicating a potential for accessing early diagnosis of NPC. Notably, BF, with a specificity of 100%, demonstrated better performance compared to IVIM in distinguishing malignant lesions from healthy tissue.

## Background

Nasopharyngeal carcinoma (NPC) is prevalent among the population of south-east Asians descent, including Chinese. A combination of radiation therapy and chemotherapy (also known as chemoradiation) is the primary treatment approach for NPC. However, survival rates are different in patients with different cancer stages; the 5-year overall survival rate is close to 100% in stage I patients, while it is only 70.5% in stage IV patients [1]. Therefore, early diagnosis of NPC is crucial to improve patients’ survival.

Nasopharyngoscope and biopsy are common diagnostic tools for NPC patients in an early stage; these patients usually show obscure or atypical symptoms. Yet, both endoscope examination and biopsy are invasive techniques that cause discomfort. In addition, many neglect tumors lie in submucosal regions [2]. Magnetic resonance imaging (MRI) is the optimal imaging method for tumor diagnosis and staging in NPC due to excellent soft-tissue contrast [3]. Stage T2 to T4 NPCs are normally associated with larger tumor volume due to fat space, surrounding muscle and skull base invasion, leading to higher diagnostic accuracy. In stage T1 NPC patients, the tumor is confined to the pharyngonasal cavity; thus, it is extremely challenging to differentiate malignant from benign lesions. Generally, an enhanced MR scan can significantly improve the diagnostic accuracy of NPC and can help distinguish stage T1 NPC from benign hyperplasia [4–7]. However, intravenous injection of gadolinium-based contrast agents is required for enhanced MR acquisition which can lead to allergy, nephrogenic systematic fibrosis, or gadolinium deposition in the brain and cause additional expenditure. Therefore, the enhanced MR examination is not suitable for patients with a contraindication to contrast medium.

Conventional non-enhanced MR scan has limited clinical diagnostic value due to the lack of signal intensity contrast between tumor and benign mucosa or peripheral muscle on MRI T1WI and T2WI. However, King et al showed that for normal or probably benign hyperplasia demonstration of the nasopharyngeal wall or adenoid on MRI, the contrast was not superior to plain scan used for detection of more cancers [8]. Consequently, it was worth exploring the value of non-enhanced MRI on the diagnosis of NPC in the early stage. Besides conventional morphology-based MRI, ASL and IVIM can be used to measure the blood perfusion and tumor microstructure using water molecule diffusion non-invasively. Nowadays, 3D-ASL, which is based on fast spin-echo (FSE) sequence and spiral K space acquisition technology, provides images with less magnetic-sensitive artifacts from the skull base, higher spatial resolution and signal-to-noise ratio (SNR). Regarding IVIM, studies revealed significant diffusion and perfusion difference between NPC and benign enlarged adenoids [9], and also between T1 stage NPC with benign hyperplasia [10]. Yet, the value of ASL on early-stage NPC remained unclear. So far, only one study reported on IVIM for the diagnosis of early NPC; however, the study included a relatively small sample size [10].

The aim of this study was to compare the perfusion and diffusion properties between stage T1 NPC and nasopharyngeal mucosa of healthy controls (HC) obtained by ASL and IVIM.

## Materials and methods

### Patients

Our institutional review board approved this prospective study. Informed consent was obtained from all the participants before the MRI examination.

From May 2015 to January 2018, 136 consecutive patients with NPC underwent an MRI scan, including both ASL and IVIM series, before undergoing nasopharyngoscopy and biopsy. Tumor stage was classified according to the 8th edition of the AJCC staging system [11]. Exclusion criteria were: (1) tumor in stage T2-T4 (*n* = 89); (2) thickness of mucosa smaller than 0.5 cm (*n* = 2). Eventually, 45 patients (35 men and 10 women) with a median age of 48 years (range: 27 to 71 years) were included in the study. According to the pathology of biopsies, 29 cases were identified as an undifferentiated type of non-keratinizing carcinoma (64.44%, 29/45) and 16 cases with the differentiated type of non-keratinizing carcinoma (35.56%, 16/45).

Besides, 32 healthy volunteers were recruited from November to December 2017. The inclusion criteria were: (1) without a history of cancer; (2) no symptom of nasopharyngeal lesions; (3) underwent MRI examination, including both ASL and IVIM series; (4) no suspected tumors in nasopharynx on the first and follow-up (at least 1 year) MRI examination. Exclusion criteria were: (1) thickness of mucosa smaller than 0.4 cm (*n* = 0). (2) poor image quality, such as obvious distortion on DWI due to frequent swallowing (*n* = 1). Finally, 31 HC (15 men, 16 women) with a median age of 38 years (range: 26–65 years) were recruited.

### MR acquisitions

All the MR acquisitions were performed on a 3.0 T whole-body MR system (Discovery MR 750, GE) with an 8-channel head and neck phase array coil. Conventional non-enhanced series were performed, including axial fast spin-echo (FSE) sequence T1WI (TR/TE = 494 ms/13.63 ms, slice thickness/slice gap = 5 mm/1 mm); axial fast recovery fast spin echo (FRFSE) T2WI with iterative dixon water-fat separation with echo asymmetry and least-squares estimation (IDEAL) (TR/TE = 4000 ms/85 ms, slice thickness/slice gap = 5 mm/1 mm, field of view [FOV] = 26 cm, scan range: from the bottom of frontal sinus to oral pharynx) (also known as the conventional T2WI); FSE T1WI sagittal (TR/ TE = 418 ms/12.35 ms, slice thickness/slice gap = 4 mm/ 0.4 mm); and DWI (SE-EPI, 4000 ms /51 ms, slice thickness/slice gap = 5 mm/1 mm, b value = 800 s/mm^2^).

Pseudo-continuous ASL (pCASL) sequence with a 3D fast spin-echo (FSE) spiral acquisition was performed, with the following parameters: axial, NEX = 3, bandwidth = 41.67, thickness = 3 mm, slice gap = 0 mm, ETL = 21, number of slice = 30, FOV = 24 cm, matrix = 288 × 192, TE = 11.1 ms, PLD 1025 ms, TR/TA = 4326 ms/262 s, and scan range: from the bottom of frontal sinus to oral pharynx. The labeling slab was automatically placed at 2.2 cm below the imaging plane. After that, another axial FRFSE T2WI series was performed with a uniform scan range as that of ASL (thickness/ slice gap of 3 mm/0 mm, called as ASL matched T2WI series), which could be fully integrated with ASL, and used to draw ROI during ASL data analysis.

IVIM series was performed using the same spatial coverage to that of conventional T2WI, with the parameters as follows: SE-EPI, TR/TE = 3500 ms/70 ms, slice thickness = 5 mm, slice gap = 1 mm, FOV = 26 cm, bandwidth = 250 Hz/pixel, matrix = 128 × 128, parallel imaging factor = 2, TA = 6 min, and 13 b values (NEX): 0 (1), 10 (2), 25 (2), 50 (2), 75 (2), 100 (1), 150 (1), 200 (1), 400 (1), 800 (4), 1000 (6), 1200 (6), and 1500 (6) s/mm^2^. Different NEX was assigned to different b values taking consideration of the SNR and the total scan time. The conventional T2WI could be completely integrated with IVIM images and used as the main reference to draw ROI when processing IVIM data. Non-enhanced series was accomplished with the same parameters for all patients and healthy volunteers. The enhanced series was not analyzed in this study because it was only conducted on patients and may cause diagnosis and measurement bias.

### Data analysis

The primary tumors were delineated by two independent observers with 5 years (XL) and 16 years (XY) of experience in tumor imaging diagnosis, respectively. To prevent ROI delineation bias, both observers were blinded to the information of either patients or healthy volunteers. Measurements were obtained using Functool software on the vendor-supplied Advantage Workstation (ADW 4.6 version, GE, US). Firstly, the slice with the thickest mucosa in HC or on the slice presenting the largest area of tumor in NPC patient was selected. Then, the region of interest (ROI), including all the mucosa tissue or the entire tumor lesion, was drawn. Other image contrasts, such as T1WI, T2WI were used as a reference to distinguish the tumor from healthy volunteers and to avoid obvious necrosis and artifacts. Another researcher (ML) checked the ROI. If the reports were inconsistent, the two observers were asked to re-draw the ROI.

The mucosal thickness and area of nasopharynx were measured on this slice by one observer (XY). The thickness of nasopharynx mucosa or tumor was the maximum diameter perpendicular to the nasopharyngeal wall, and the area of nasopharynx mucosa or tumor were delineated, including all the mucosal tissue or tumor at this slice.

### ASL data analysis

The data were processed as follows: ASL series were imported to Functool Software (named “ASL”); ASL images were integrated with ASL matched T2WI series; the image contrast was adjusted to draw the ROI on T2WI series (ROI could be copied automatically on ASL image and BF map); BF value was ultimately acquired. BF was calculated according to the described equation [12]:

$$ BF=\frac{6000\bullet \lambda \bullet \left({SI}_{control}-{SI}_{label}\right)\bullet {e}^{\frac{PLD}{T_{1, blood}}}}{2\bullet \alpha \bullet {T}_{1, blood}\bullet {SI}_{PD}\bullet \left(1-{e}^{-\frac{\tau }{T_{1, blood}}}\right)}\left[ mL/100g/\mathit{\min}\right] $$

where λ represents the tumor-tissue /blood partition coefficient in mL/g (0.9 mL/g); SI_control_ and SI_label_ refer to the time-averaged signal intensities in control and label images, respectively; T_1,blood_ is the longitudinal relaxation time of blood in seconds (1650 ms); α represents the labeling efficiency (0.85); SI_PD_ is the signal intensity of a proton density-weighted image; τ is the label duration, and PLD indicates the post-labeling delay time. A factor of 6000 converts the units from mL/g/s to mL/(100 g)/min, which is customary in physiological literature.

### IVIM data analysis

IVIM data was analyzed using Functool software (MADC). Due to possible distortions resulted from the SE-EPI sequence, image at b value = 1000 s/mm^2^ was selected to draw ROI with the conventional T2WI series as the main reference.

Apparent diffusion coefficient (ADC) was achieved by the mono-exponential model (MEM) using the following equation:

$$ S(b)/{S}_0=\mathit{\exp}\left(-b\times ADC\right) $$

Bi-exponential model (BEM) allows the separation of a “microvascular” compartment (perfusion) and a “nonvascular” compartment (diffusion) using the following equation:

$$ S(b)/{S}_0=\left(1-f\right)\ \mathit{\exp}\ \left(-b\times D\right)+f\ \mathit{\exp}\left(-b\times D\ast \right) $$

where S(b) and S_0_ represent the mean signal intensity with or without diffusion gradient b, respectively; f is the microvascular volume fraction representing the fraction of diffusion linked to microcirculation; D refers to the pure molecular diffusion; D* is the pseudo-diffusion coefficient.

### Statistical analysis

Statistical analyses were performed using SPSS Statistics Version 25.0 (v 25.0 IBM Company Armonk, NY, USA), MedCalc 13.0.4.0 (MedCalc, Mariakerke, Belgium) and Graphpad Prism 5.0 (Graphpad Software. San Diego, CA). Inter-observer consistency was investigated using a two-way random intra-class correlation (ICC), which was interpreted as: < 0.5, poor; 0.5–0.75, moderate; 0.75–0.9, good; > 0.90, excellent [13]. After that, the averages of the measurements from two observers were used for further comparison. Mann-Whitney test was used to compare the difference of the ASL and IVIM derived parameters between tumors and nasopharyngeal mucosa of HC since all the metrics were not in normal distribution as proved by Kolmogorov–Smirnov test. The areas under curve (AUC) of the receiver operating characteristic (ROC) curve was calculated and compared for the significant parameters using Delong’s test, and to determine the diagnostic cutoff and further efficiency. Spearman’s correlation analysis was performed to investigate the relationship between BF and IVIM derived perfusion parameters (D* and f). *P*-value < 0.05 was considered to be statistically significant.

## Results

### Comparison between T1 stage NPC and nasopharyngeal mucosa of HC

There was no significant difference in age between NPC and HC groups (*P* > 0.05). The median thickness and area of tumors were 13.67 mm (range: 6–25 mm) and 214.24 mm^2^ (range: 77–429 mm^2^), respectively. The median thickness and area of mucosa on HC was 7 mm (range: 4–20 mm) and 120 mm^2^ (range: 33–351 mm^2^), respectively.

**Fig. 1:**
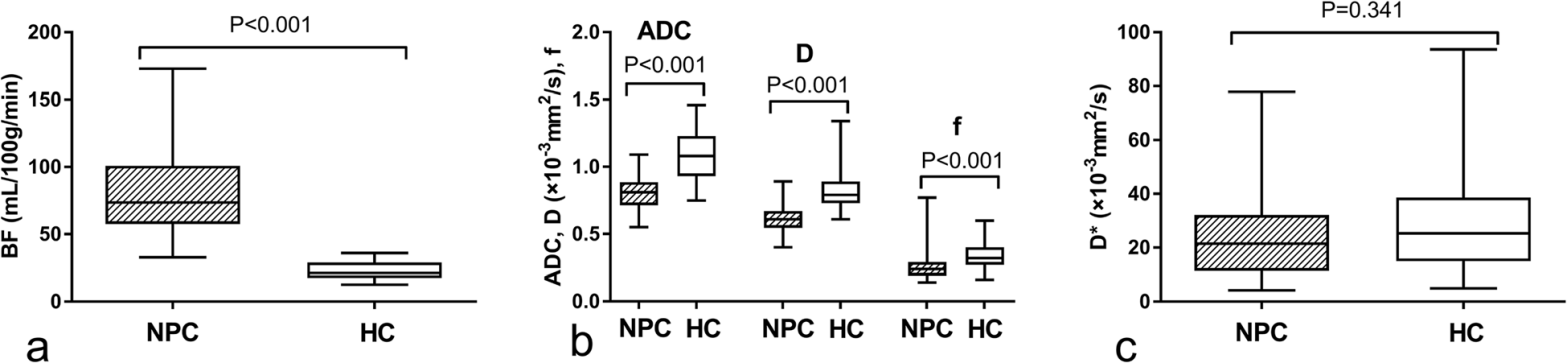
Box-plots of comparisons between two groups. NPC tumor in stage T1 had significantly higher BF, lower ADC, D, and f than those of nasopharyngeal mucosa of HC. There was no inter-group statistical difference in D*. NPC = nasopharyngeal carcinoma, HC = healthy controls

BF and IVIM derived parameters were shown in Table 1. Good to excellent consistency (ICC: 0.893–0.994) was observed between two observers. Compared with nasopharyngeal mucosa of HC, NPC tumors in T1 stage had significantly higher BF, lower ADC, D, and f (*P*: <0.001). There was no inter-group statistical difference in D* (Table 1, Fig. 1).

**Table 1:** Inter-observer consistency and comparisons of the absolute values of the parameters

	ICC^§^ (95% confidence interval)	Tumor of T1 stage NPC	Mucosa of healthy control	*P*	z	AUC (95% confidence interval)
BF (mL/min/100 g)	0.994 (0.991–0.996)	81.85 ± 32.46	22.89 ± 6.84	**<0.001**	7.309	0.996 (0.944–1.000)
ADC (×10^−3^ mm^2^/s)	0.946 (0.915–0.966)	0.81 ± 0.12	1.08 ± 0.19	**<0.001**	−5.808	0.895 (0.803–0.953)
D (×10^−3^ mm^2^/s)	0.893 (0.831–0.932)	0.61 ± 0.10	0.83 ± 0.15	**<0.001**	−6.226	0.921 (0.836–0.970)
D^*^ (×10^−3^ mm^2^/s)	0.894 (0.832–0.933)	26.21 ± 19.09	28.49 ± 18.50	0.341	−0.951	–
f	0.900 (0.842–0.936)	0.27 ± 0.11	0.34 ± 0.11	**<0.001**	−3.525	0.742 (0.628–0.835)

^§^ ICC is the abbreviation of Intra-class correlation

All the values were calculated as the mean value of the measurements by two observers

**Fig. 2:**
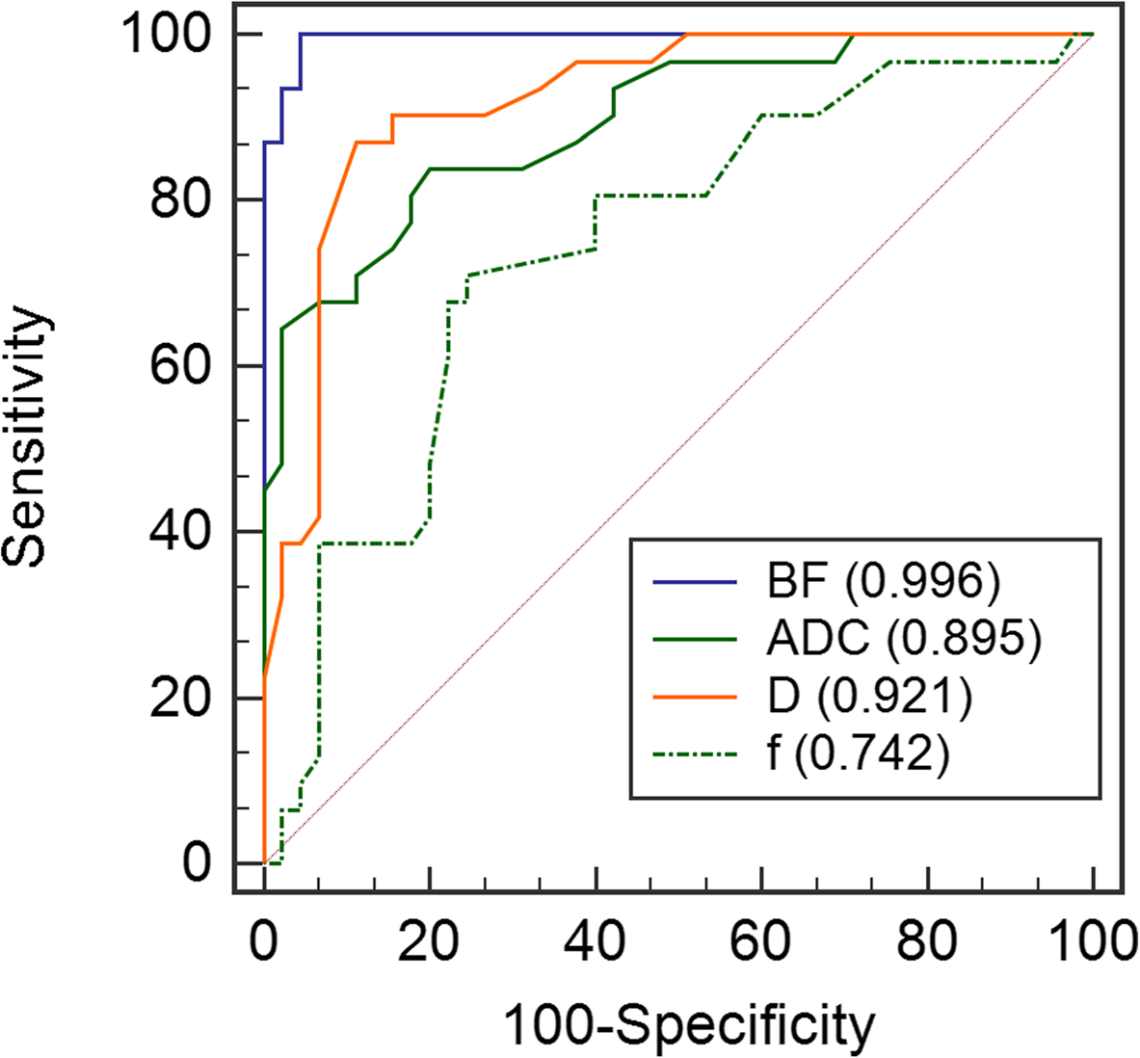
ROC curves by the parameters with significance in differentiating stage T1 NPC from nasopharyngeal mucosa of HC. AUCs of the parameters are shown in parentheses

Further ROC comparison of the parameters showed that AUC of BF was significantly higher (0.996) than that of ADC (0.895), D (0.921) and f (0.742) (*P* = 0.006, 0.017 and < 0.001, respectively). AUC of f was lower than that of ADC and D (*P* = 0.022 and 0.007, respectively), and there was no statistical difference of AUC between ADC and D (*P* = 0.314; Fig. 2). BF cutoff was set at larger than 36 mL/100 g/min to achieve the sensitivity (95.56%, 43/45), specificity (100%, 31/31), and accuracy (97.37%, 74/76) on differentiating the NPC in T1 stage from nasopharyngeal mucosa of HC, respectively.

**Fig. 3:**
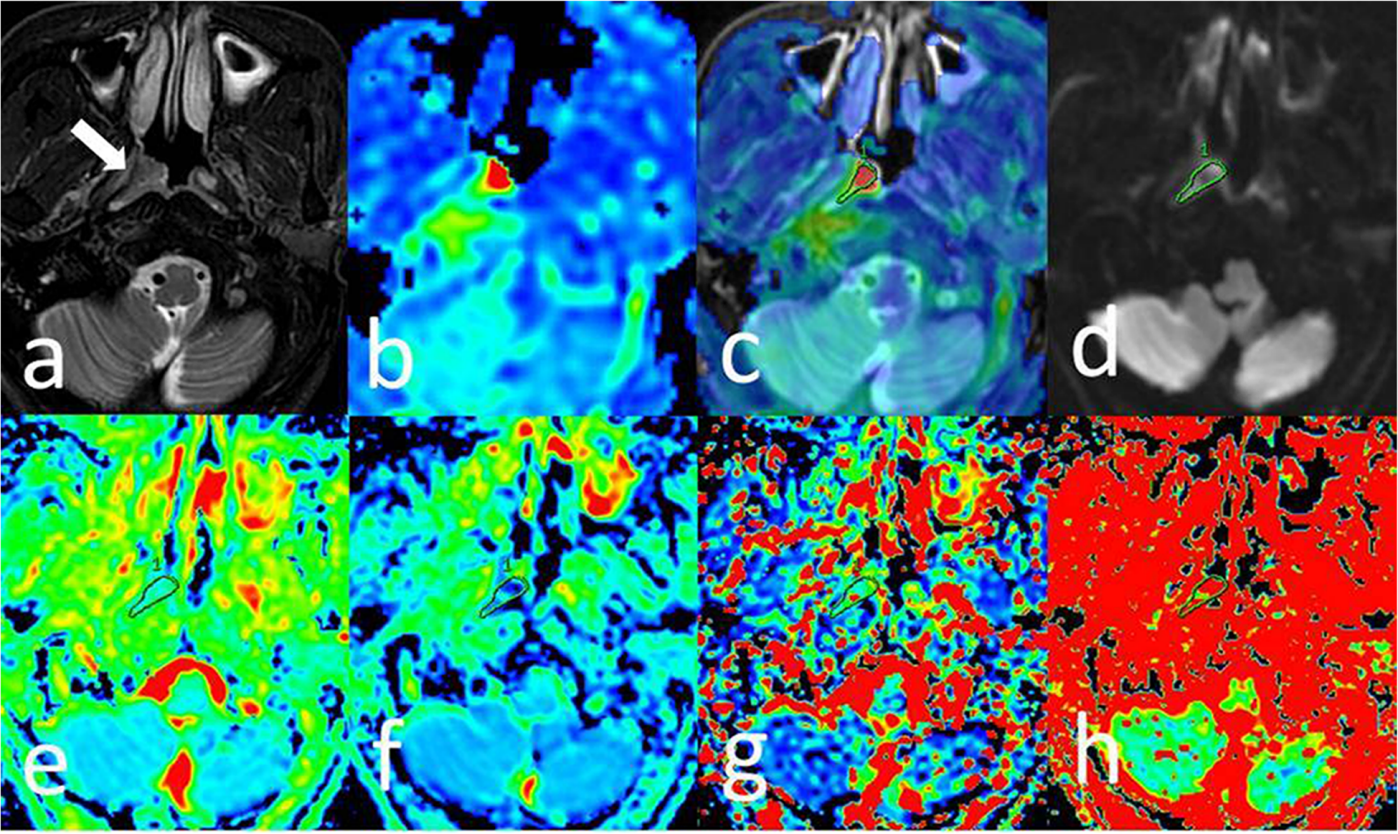
M50, differentiated type of non-keratinizing nasopharyngeal carcinoma. **a** T2WI/IDEAL showing a mass (arrow) invasion in the right pharyngeal recess and posterior nasopharyngeal wall. **b** BF map showing relatively higher perfusion of tumor compared to surrounding tissues. **c** Merged image (image a and b) showing ROI of the tumor on ASL and acquired BF of the tumor as 183.88 mL/min/100 g. IVIM image with b value = 1000 s/mm^2^ (d) showed diffusion restriction in the tumorous area. ADC (0.874 × 10^− 3^ mm^2^/s), D (0.527 × 10^− 3^ mm^2^/s), D* (15.6 × 10^− 3^ mm^2^/s) and f (0.28) (e-h) were obtained

**Fig. 4:**
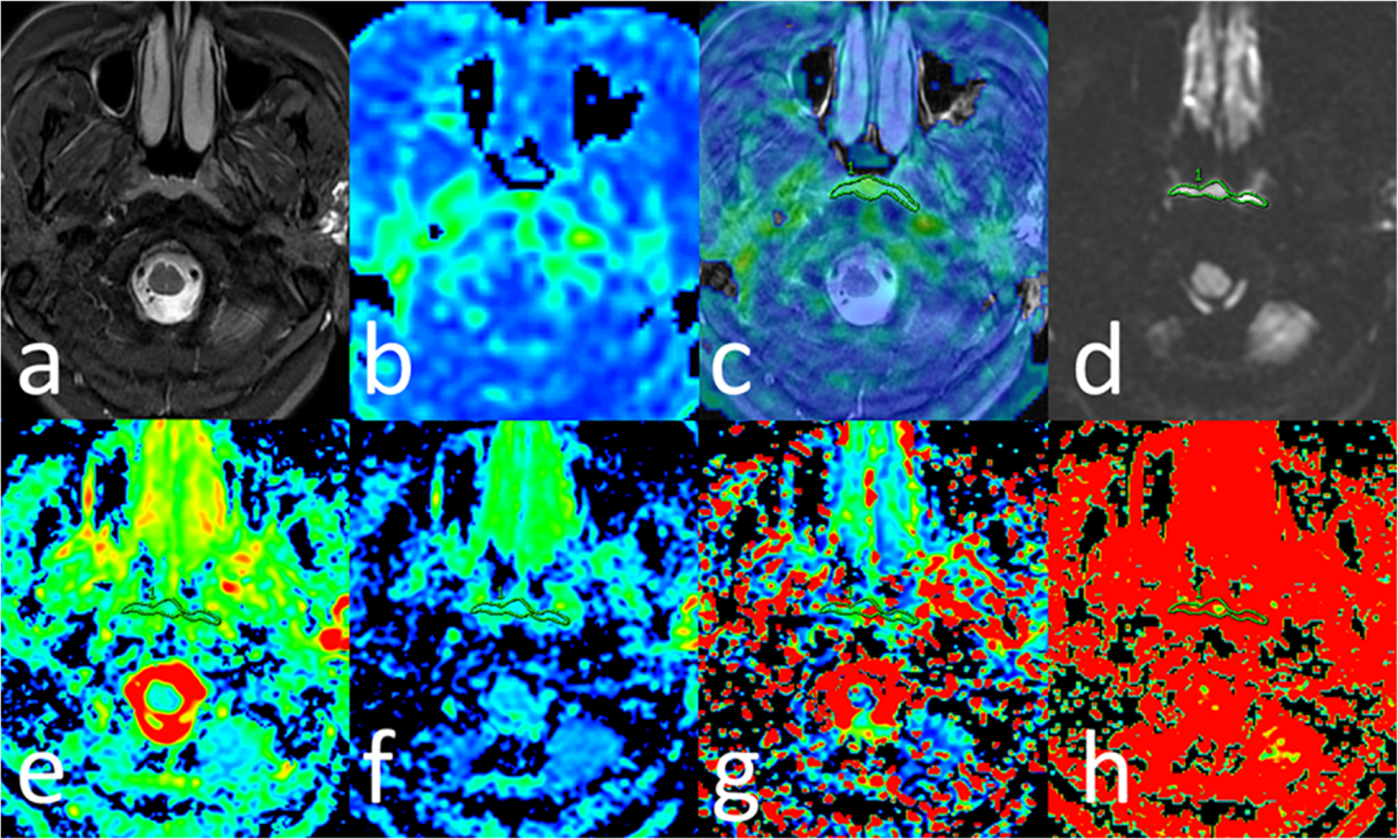
F58, healthy control. **a** T2WI/IDEAL showing the symmetrical bilateral nasopharyngeal mucosa thickening. **b** BF map showing relatively lower perfusion of tumor compared to surrounding tissues. **c** Merged image (image a and b) showing ROI of mucosa on ASL and acquired BF of nasopharyngeal mucosa as 32.37 mL/min/100 g. **d** IVIM image with b value = 1000 s/mm^2^ showing the diffusion restriction in the tumor area. **e-h** ADC (1.06 × 10^− 3^ mm^2^/s), D (0.80 × 10^− 3^ mm^2^/s), D* (34.5 × 10^− 3^ mm^2^/s), and f (0.30) were obtained

Representative image examples of NPC in the T1 stage and HC were shown in Figs. 3 and 4.

### Correlation between parameters of ASL and IVIM

**Fig. 5:**
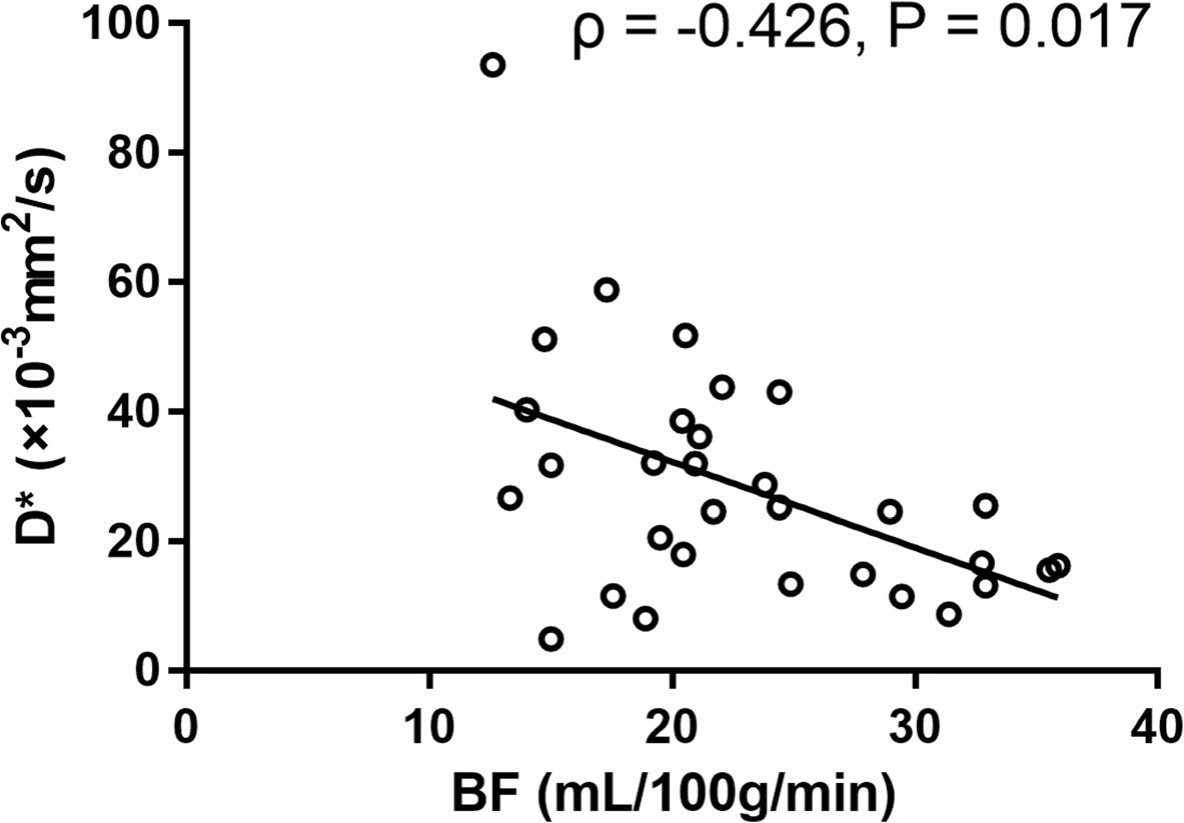
Scatterplot of BF and D*. BF demonstrated a moderate negative correlation with D* on the group of healthy control

On the group of HC, BF demonstrated a moderate negative correlation with D* (ρ [Spearman correlation coefficients] = − 0.426, *P* = 0.017), and no correlation with f. On the NPC group, no correlation was found between BF and D* or f (Table 2, Fig. 5).

**Table 2:** Correlation between BF and parameters of IVIM

		D^*^ (×10^− 3^ mm^2^/s)		f	
		ρ^§^	P	ρ	P
Tumor of T1 stage NPC (*n* = 45)	BF (mL/min/100 g)	−0.050	0.745	0.203	0.180
Mucosa of healthy control (*n* = 31)	BF (mL/min/100 g)	**−0.426**	**0.017**	−0.354	0.051

^§^ ρ represents Spearman correlation coefficients

## Discussion

In the current study, the perfusion and diffusion properties derived by ASL and IVIM significantly differed between the NPC and HC, especially for BF values, which might work as an effective marker to improve the accuracy of non-enhanced MR scan on diagnosing early-stage NPC.

ASL, processed by a simpler model, is capable of generating a single parameter named BF value with a good reproducibility [14, 15], which was also consistent with our study results revealing the highest inter-observer consistency via BF. BF derived from ASL can be used to evaluate the perfusion characteristics of the tumor quantitatively. Previous studies have revealed slight to moderate positive correlation between BF and dynamic contrast-enhanced MRI (DCE-MRI) quantitative indices (K^trans^, K_ep_) in NPC tumors or semi-quantitative indices (positive enhancement integral, the maximum slope of increase, the maximum slope of decrease), and moderate negative correlation between BF and time to peak while taking all NPC and non-NPC areas into account [16, 17]. So far, only a few studies applied ASL on NPC. Xiao et al revealed higher BF on tumor as compared to the contralateral side of the tumor (64.3 ± 21.0 vs. 29.5 ± 9.7 mL/min/100 g), which was consistent with our study [17]. Moreover, other studies showed BF could help delineating the tumor extent by the fusion image of the BF map with T2WI [18] and evaluating clinical stages with moderate positive correlations to T stage and AJCC stage [19]. For head and neck tumors, studies suggested that ASL could assist the diagnosis and differentiation of pathological types of tumors [20, 21], monitor the effect of head and neck tumor before and after non-surgical treatment, and evaluate the potential existence of residual tumors [22].

For IVIM, both ADC and D were able to describe the degree to which the tissue diffusion was restricted. Generally, malignant tumor cell growth results in an elevated level of cellular density and shrinkage of extracellular space, which leads to lower ADC and D values compared to benign tumors. Ai et al found that early-stage NPC had lower D, ADC_0–1000_, and ADC_300–1000_ than benign hyperplasia [10]. Another study showed variation in D values when differentiating NPC from benign enlarged adenoids [9]. The above data were consistent with our results; both the ADC and D exhibited significant statistical differences for lower value in NPC than benign nasopharyngeal mucosa.

Previous studies revealed that perfusion related parameters of IVIM had a significant correlation with DCE-MRI parameters, i.e., both f and D* were positively correlated with K^trans^, K_ep_ [23]; only f was positively correlated with enhancement amplitude and ratio on DCE-MRI [24]. Ai et al reported a higher D* of early-stage NPC than that of benign hyperplasia (32.66 ± 4.79 vs. 21.96 ± 5.21 × 10^− 3^ mm^2^/s) [10]. Contrary, Zhang et al [9] observed lower D* (48.33 ± 17.42 vs. 152.96 ± 27.41 × 10^− 3^ mm^2^/s) but higher f (26.72 ± 4.9% vs. 16.44 ± 2.01%) in benign enlarged adenoids compared to NPC. In this study, no difference in D* was found between the two groups. Furthermore, D* value of HC in our results were similar to benign hyperplasia found by Ai et al [10] but smaller than that of benign enlarged adenoids achieved in the study performed by Zhang et al [9]. It is possible that benign enlarged adenoids accompanied by increasing blood perfusion might be regarded as benign lesions instead of normal nasopharyngeal mucosa. Furthermore, Federau et al [25] indicated that D* in the healthy human brain might be influenced by cardiac cycles and pulsatile flow, which results in significantly higher D* value in systole. A wide range of D* values was reported (16.0 to 143.914 × 10^− 3^ mm^2^/s) on NPC in the literature [24, 26]. Therefore, a study on the reliability and repeatability D* is required in the future. Regarding the f value, one study showed no statistical difference between early-stage NPC and benign hyperplasia [10]. In this study, although NPC was associated with increased capillary perfusion, decreased f was found in NPC compared to benign nasopharyngeal mucosa. This result was also consistent with previous studies on other types of tumors [27–29]. It is considered that f is associated with TE (especially for tissue with considerably shorter transversal relaxation times than blood), the range of b-values (f value was significantly increased in tumors with b-values below 750 s/mm^2^), and own tissue features (neovascularization of tumor vs. microvasculature of benign nasopharyngeal mucosa) [28, 30–32].

Several studies have investigated the correlations between BF and perfusion parameters of IVIM; yet, contradictory conclusions were obtained. For instance, Shen et al [33] showed that BF had a positive correlation with D* and f in brain gliomas (r = 0.486, 0.560, respectively). Dolgorsuren et al [34] also showed a moderate correlation between BF and f (r = 0.414) in brain tumors, but no correlation between BF and D*. However, the study on brain glioma by Lin et al [35] showed that BF had a negative correlation with f (r = − 0.619), whereas no correlation was observed between BF and D* in both tumor and white matter. The inconsistency may be interpreted as the result of different choices of b values, parameters of the DWI scheme, and time of echo. Our study showed that only the BF of HC had a moderate negative correlation with D*, while no correlation was found between BF and D* or f in NPC. Although both BF and IVIM can reflect perfusion of tissue, it is considered that they reflect different characteristics of perfusion. Since BF could reflect the blood flow, while D* maybe contain more information on permeability [34], the correlation between BF and D* still needs to be confirmed by larger samples. One study considered that BF is more suitable for the evaluation of perfusion in brain tumors since BF by ASL showed a stronger correlation with the parameters of dynamic susceptibility contrast (relative blood volume [rBV] and corrected relative blood volume [crBV]) than D* and f by IVIM [34]. In this study, based on AUC, BF was the most powerful parameter with a statistical difference to other parameters, which indicated that BF could be applied as a reliable marker to distinguish NPC in the T1 stage from benign nasopharyngeal mucosa.

Post-labeling delay time (PLD) is defined as the waiting time for blood to transit from the labeling site to the imaging volume, which serves as an important factor in ASL protocol and impacts the measured BF level. Under short PLD, the labeled blood may not fully enter the tissue, whereas prolonged PLD may reduce the SNR, both of which may affect the accuracy of BF. Previous studies on NPC [16, 18] applied ASL with PLD of 1025 ms, 1525 ms, and 2025 ms. The optimal selection of PLD is 1025 ms due to its best performance on evaluating perfusion and delineating the volume of NPC, which was also similar to previous studies on malignant tumors of nasal cavity and neck with PLD of 1280 ms [20, 36].

### Limitations

This study has a few limitations. Firstly, only the T1 stage NPC was included, which resulted in a relatively small sample size. More cases are needed to confirm these findings. Secondly, the healthy volunteers did not undergo nasopharyngoscopy/biopsy; thus, there may be potential asymptomatic chronic inflammation within the control group. Thirdly, the ROI cannot be replicated between the two measurement software. Yet, considering the difference (i.e., imaging principle, distortion) between the two kinds of functional series, the same ROI on both software can lead to the incorrect delineation of the tumor area. Therefore, two observers were asked to choose the slice with a maximum area of the primary tumor or the slice with the thickest mucosa of HC based on T2WI and to draw the ROI according to the signal intensity of each series to reflect its characteristics. Lastly, we set PLD at 1025 ms according to previous studies [16, 18], without PLD optimization. More PLD selection will be applied in future research with comparison and clinical relevance.

## Conclusion

ASL and IVIM could be used to evaluate the perfusion and diffusion of NPC in the T1 stage, and become a complementary tool for conventional structural MRI for the early-stage NPC diagnosis in clinical practice. Also, compared with the parameters derived from IVIM, BF of ASL resulted as the most promising marker with the specificity of 100%.

## Data Availability

Not applicable.

## Abbreviations

NPC: Nasopharyngeal carcinoma
ASL: Arterial spin labeling
PLD: Post-labeling delay
BF: Blood flow
DWI: Diffusion-weighted imaging
IVIM: Intravoxel incoherent motion
ADC: Apparent diffusion coefficient
D: Pure molecular diffusion
D*: Pseudo-diffusion coefficient
f: Perfusion fraction
